# Asiatic acid, a triterpene, inhibits cell proliferation through regulating the expression of focal adhesion kinase in multiple myeloma cells

**DOI:** 10.3892/ol.2013.1597

**Published:** 2013-10-01

**Authors:** JUNLI ZHANG, LISHA AI, TINGTING LV, XUDONG JIANG, FANG LIU

**Affiliations:** 1 Institute of Hematology, Union Hospital, Tongji Medical College, Huazhong University of Science and Technology, Wuhan, Hubei 430022, P.R. China; 2 Department of Hematology, Xiangyang Center Hospital, Xiangyang, Hubei 441021, P.R. China; 3 Department of Hematology, The First Affiliated Hospital of Shantou University Medical College, Shantou, Guangdong 515041, P.R. China

**Keywords:** asiatic acid, multiple myeloma, focal adhesion kinase, proliferation inhibition, cell cycle arrest

## Abstract

The aim of the present study was to investigate whether asiatic acid (AA), a pentacyclic triterpene derived from *Centella asiatica*, exerts anti-proliferative effects on multiple myeloma RPMI 8226 cells and to determine the molecular mechanism underlying the anticancer action of AA. The study sought to analyze the potential role of AA on the proliferation of the RPMI 8226 cells using a 3-(4,5-dimethyl-2-thiazolyl)-2,5-diphenyl-2H-tetrazolium assay. Cell cycle arrest was detected by flow cytometry, and the expression levels of focal adhesion kinase (FAK) in the myeloma cells induced by AA were analyzed using the western blotting and immunoprecipitation methods. The results indicated that AA significantly inhibited cell proliferation in a time- and dose-dependent manner and led to G_2_/M phase arrest at concentrations of 35 and 40 μmol/l in the RPMI 8226 cells. The expression levels of FAK and p-FAK were distinctly decreased following AA treatment (at the concentration of 40 μmol/l) for 24 h compared with that of the control groups. Taken together, these results demonstrated that AA was able to regulate cell cycle progression in RPMI 8226 cells, thereby significantly inhibiting cell growth. Furthermore, AA decreased the expression levels of FAK, indicating that the antitumor mechanism of AA may be associated with the inhibition of signal transduction mediated by FAK.

## Introduction

Multiple myeloma (MM) is a plasma cell malignancy that is considered to be the second most common hematological cancer in the world (1). The remission rate of MM remains low due to the complex pathogenesis and multidrug resistance. Newer chemotherapeutic regimens and high-dose chemotherapy have increased the response rate in myeloma. However, the unpleasant side-effects, including hepatotoxicity, cardiotoxicity, hematotoxicity and infection, restrict their clinical efficacy. In recent studies, investigators have recognized the potential use of natural products as potent chemotherapeutic drugs for MM to improve the therapeutic efficacy and also reduce the side-effects (2,3). For instance, oridonin, an active diterpenoid compound isolated from *Rabdosia rubescens*, simultaneously induces the apoptosis and autophagy of human MM cells (4).

Asiatic acid (AA), a pentacyclic triterpenoid derived from the tropical medicinal plant *Centella asiatica* (Apiaceae family), has a wide variety of biological activities. For a long period of time, AA was mainly believed to be responsible for wound healing, protective activities against UV-induced photo aging, glutamate- or β-amyloid-induced neurotoxicity and hepatofibrosis (5–9). Recently, the apoptosis-inducing activity of AA in various cancer cells has aroused the attention of investigators (10). For example, AA has been successively reported to possess strong cell growth inhibition in hepatoma, breast cancer, melanoma, glioblastoma and gastrointestinal tumor cells (11–15). However, the effects of AA on hematological malignant cells remain unclear. Thus, in the present study, AA was first identified to inhibit cell proliferation through the arrest of RPMI 8226 cells at the G_2_/M phase, whereas little is known about the mechanism of AA-induced anti-myeloma action. A previous study determined that celastrol, which is also a triterpene, exerts antitumor activities accompanied by the reduced phosphorylation of focal adhesion kinase (FAK) (16). Previous studies also showed that phosphatase and tensin homolog deleted on chromosome 10 prevented the metastasis of myeloma cells by downregulating the activity of the FAK/matrix metalloproteinase signaling pathway. FAK is a non-receptor tyrosine kinase that modulates cell adhesion, movement and survival, which may be associated with disease progression, extramedullary infiltration and the apoptosis of MM cells (17,18). Earlier studies have indicated that the suppression of FAK expression, caused by interrupting the nuclear factor κB pathway, provided a potential molecular target in MM (19,20). Hence, in conjunction with these findings, we speculate that the underlying mechanism of the anti-proliferation function of AA may be through the inhibition of FAK expression in MM cells.

## Materials and methods

### Main reagents

AA (molecular formula, C_30_H_48_O_5_; molecular weight, 488.7 Da), 3-(4,5-dimethyl-2-thiazolyl)-2,5-diphenyl-2H-tetrazolium (MTT), dimethyl sulfoxide (DMSO) and propidium iodide (PI) were purchased from Sigma-Aldrich (St. Louis, MO, USA). A 50-mmol/l AA stock solution was prepared in DMSO, stored at −20°C as small aliquots and then thawed prior to use. RPMI-1640 media and phosphate-buffered saline (PBS) were purchased from Invitrogen (Carlsbad, CA, USA). Fetal bovine serum (FBS) products were purchased from Hangzhou Sijiqing Biological Engineering Materials Co., Ltd. (Hangzhou, Zhejiang, China). Lymphoprep Ficoll was purchased from Axis-Shield (Oslo, Norway). The PI reagent kit was purchased from Nanjing Key-Gen Biotech Co., Ltd. (Nanjing, Jiangsu, China).

### Cell culture

The RPMI 8226 cells had been stored long-term and passaged in the Institute of Hematology, Huazhong University of Science and Technology (Wuhan, China). The RPMI 8226 cell line, a human factor-independent myeloma cell line, was cultured in RPMI-1640 medium supplemented with 10% FBS at 37°C in a humidified atmosphere containing 5% CO_2_. The culture medium was replaced with fresh medium every 2 to 3 days. The cells in the mid-log phase were used in the experiment. The collection of blood samples and the isolation of peripheral blood mononuclear cells (PBMCs) were performed as previously reported (21). All blood donors provided their informed consent. Briefly, the cells were used directly after isolation and stored in RPMI-1640 medium with 10% FBS, 1% penicillin/streptomycin and 1% L-glutamine (both from Invitrogen) overnight prior to incubation.

### MTT assay

The effects of AA on the proliferation of the RPMI 8226 cells were detected by MTT assay. Briefly, the RPMI 8226 cells were harvested at mid-log phase and the PBMCs were prepared as a control group. Subsequently, a 200-μl suspension of cells was seeded in 96-well plates with or without AA at various concentrations (10, 20, 30, 40, 50, 60 and 70 μmol/l) at a density of 3×10^5^ cells/well. Subsequent to incubation for a designated period of time, 20 μl MTT solution (5 mg/ml) was added, and the cells were incubated at 37°C for another 4 h. The supernatant was discarded and 150 μl DMSO was added. The plate was gently vortexed until the blue formazan crystals were fully dissolved. The absorbance (A) was read at an optical density of 490 nm using a microplate reader (Tecan Spectra; Tecan Group Ltd., Männedorf, Switzerland) and the growth inhibitory rates were calculated as follows: [1 - (A of experimental sample / A of the control sample)] × 100.

### Flow cytometric analysis

The cells in mid-log phase were divided into the control and the experimental groups, and the cell concentration of each group was 1×10^6^/ml. The RPMI 8226 cells were treated with various concentrations of AA (0, 25, 35 and 40 μmol/l), and the cell cycle was analyzed by flow cytometry (FCM). The RPMI 8226 cells were collected following treatment using EP tubes, fixed in 70% cold ethanol for 24 h, washed twice with PBS and resuspended in 440 μl PBS. A volume of 10 μl RNaseA (5 mg/ml) was added into the tube and incubated for 30 min. Subsequently, 50 μl PI was added and the cells were incubated at 4°C in the dark for another 30 min. The fluorescence intensity was detected using a flow cytometer (Becton-Dickinson, Franklin Lakes, NJ, USA) and the cell cycle was analyzed using FlowJo software (version 7.6; Tree Star Inc., Ashland, OR, USA).

### Western blot analysis

All the RPMI 8226 cells treated with AA at various concentrations for 24 h were collected and subjected to western blot analysis. The cells were lysed in a modified RIPA lysis buffer, and the protein in the supernatant was quantified using the Coomassie Brilliant Blue kit (Pierce, Rockford, IL, USA). The prepared protein samples were stored at −10°C prior to use. Next, 10% sodium dodecyl sulphate-polyacrylamide gel electrophoresis (90 μg protein per lane) was performed; the proteins were then transferred to polyvinylidene difluoride (PVDF) membranes. The membranes were blocked in PBS Tween-20 (5 g/l) containing skimmed milk (at the concentration of 50 g/l) at 4°C overnight, then washed and incubated with primary rabbit anti-human FAK polyclonal antibody. The PVDF membranes were then washed and incubated with the horseradish peroxidase-conjugated secondary antibody (Sanying Biotechnology Co., Wuhan, Hubei, China) and exposed for 2 sec using chemiluminescent autoradiography. The X-ray films were then developed.

### Immunoprecipitation

A volume of 30 μl mouse anti-human PY100 was added into 200 μg total protein and vortexed at room temperature for 1 h. Next, 50 μl protein G (Sanying Biotechnology Co.) was added into the tube for precipitation and the sample was washed. The remaining steps were in line with the western blot analysis.

### Statistical analysis

Each experiment was repeated at least three times. The data were presented as the mean ± SD and analyzed using SPSS 11.0 Statistical Software for Windows (SPSS, Inc., Chicago, IL, USA). The comparisons between each group were analyzed by t-test. Statistically significant differences were indicated by P<0.05.

## Results

### AA inhibits the proliferation of RPMI 8226 cells

**Figure 1:**
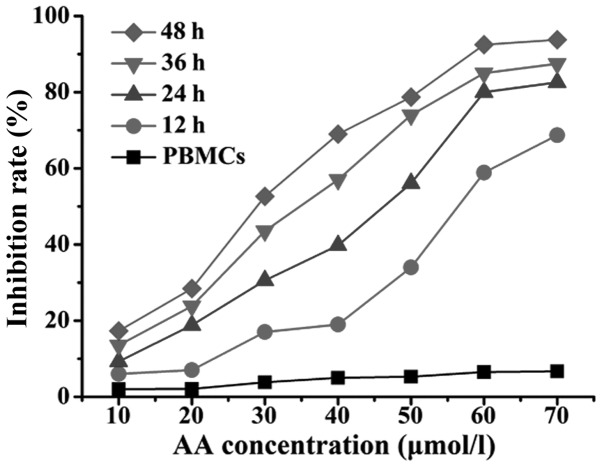
Asiatic acid (AA) at various concentrations inhibited the proliferation of RPMI 8226 cells. Cell growth inhibition of AA was determined using MTT assay. The RPMI 8226 cells were exposed to 0–70 μmol/l AA at 12 h (●), 24 h (▲), 36 h (▼), 48 h (◆), and the PBMCs (■) were also treated with various concentrations of AA. The results are expressed as the mean of at least three independent experiments.

To investigate whether AA exerted anti-proliferative effects on the MM cells, the cytotoxicity of various concentrations of AA (0, 10, 20, 30, 40, 50, 60 and 70 μmol/l) on RPMI 8226 cells for 12, 24, 36 and 48 h was detected by MTT assay. As shown in Fig. 1, the cell viability was inhibited by AA in a time- and dose-dependent manner in the RPMI 8226 groups. By contrast, rarely detectable changes were exhibited in the viability of the PBMCs. The rate of proliferative inhibition for the RPMI 8226 cells increased significantly following incubation with various concentrations of AA for 12 h (P<0.05). At the same concentration (40 μmol/l), the rate was also significantly different at various times (P<0.05). The IC_50_ of AA for the RPMI 8226 cells was 53.76±2.88, 42.25±4.57, 32.78±3.25 and 24.88±3.51 μmol/l at 12, 24, 36 and 48 h, respectively.

### AA induces cell cycle arrest in RPMI 8226 cells

**Figure 2:**
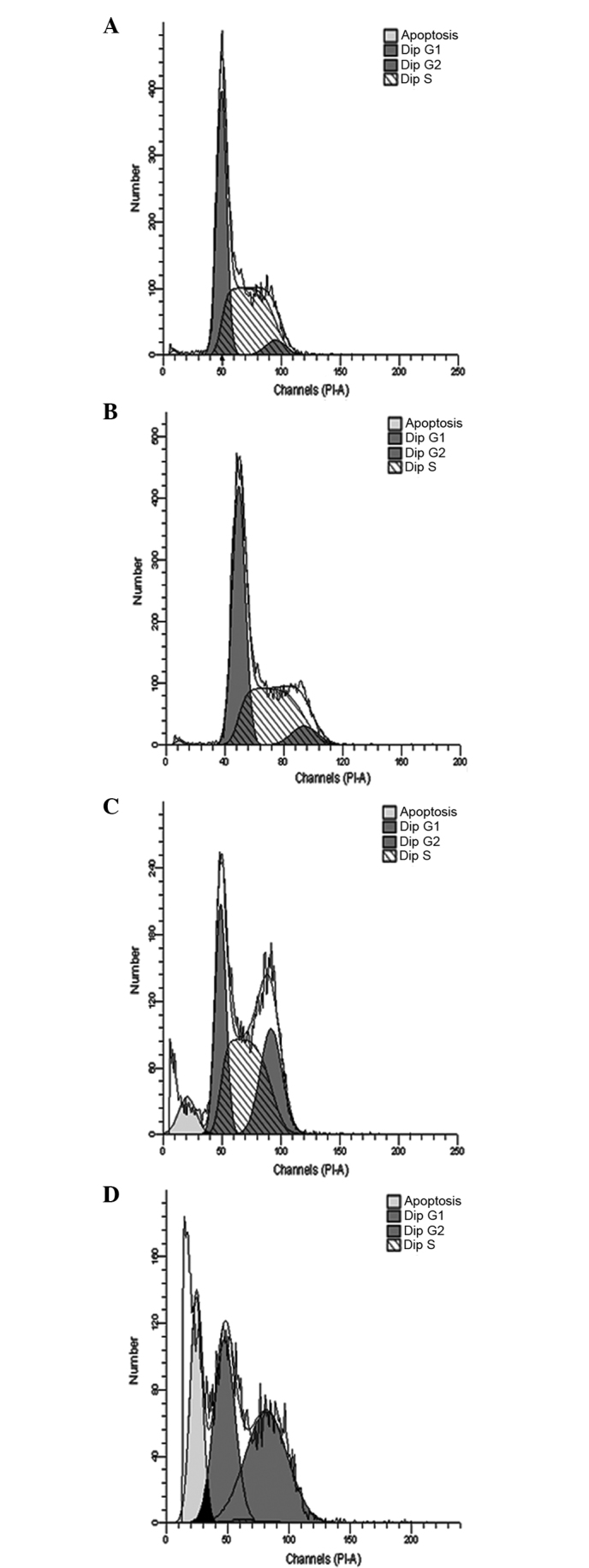
Effects of asiatic acid (AA) on cell cycle arrest in RPMI 8226 cells. Following the treatment of the cells with AA for 24 h, the cell cycle changes were analyzed by flow cytometry. The cell cycle distribution of the RPMI 8226 cells was assessed following exposure to (A) 0, (B) 25, (C) 35 and (D) 40 μmol/l AA. The results are expressed as the mean of at least three independent experiments.

Subsequent to the treatment of the RPMI 8226 cells with various concentrations of AA for 24 h, the cell cycle analysis by FCM showed that the percentage of RPMI 8226 cells in the G_2_/M phase had increased significantly at each time-point tested. The cell cycle distribution of the RPMI 8226 cells measured at various time points is shown in Fig. 2. The cells exposed to 35 and 40 μmol/l AA showed evident cell cycle arrest, with cells predominantly arrested in the G_2_/M phase. Notably, no evident changes of increased G_2_/M phase cells were noted in the groups treated with 25 μmol/l AA.

### AA decreases the expression of FAK and p-FAK

**Figure 3:**
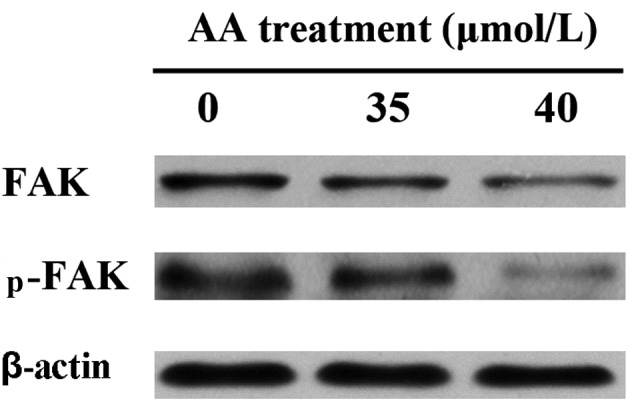
Expression levels of focal adhesion kinase (FAK) and p-FAK in asiatic acid (AA)-treated RPMI 8226 cells. The cells were incubated with 0, 35 and 40 μmol/l AA. The expression of FAK was determined by western blotting (lane 1) and p-FAK was assessed by immunoprecipitation (lane 2). The images are representative of three separate experiments.

The expression levels of FAK and p-FAK were assessed in the AA-treated RPMI 8226 cells by western blotting and immunoprecipitation. As shown in Fig. 3, the exposure of the RPMI 8226 cells to 35 and 40 μmol/l AA for 24 h resulted in a significant inhibition of FAK and p-FAK in a dose-dependent manner compared with the control group.

## Discussion

Despite gradual advancements in the understanding of drug combinations for MM, the side-effects and relatively low remission rate of chemotherapy have spurred a number of researchers to establish more effective treatment regimens by adopting novel and innovative approaches. The discovery and exploitation of active medicinal compounds from natural sources have provided alternative treatment choices for patients (22). For example, AA, a triterpene acid derived from the traditional medicinal plant *C. asiatica*, belongs to the pentacyclic triterpenoids. There have been numerous studies demonstrating the strong anti-solid tumor efficacy of AA. AA has been reported to induce apoptosis in human hepatoma, breast cancer, melanoma, glioblastoma and gastrointestinal tumors (11–15). The major findings of the present study were that AA appeared to inhibit the cell proliferation of the RPMI 8226 cells with the effective concentration being at the μmol/l level, consistent with the effective concentration level of AA in solid tumors. In addition, the marked anti-proliferative activity induced by AA occurred in a time- and dose-dependent manner, with an IC_50_ of 53.76±2.88, 42.25±4.57, 32.78±3.25 and 24.88±3.51 μmol/l in the RPMI 8226 cells at 12, 24, 36 and 48 h, respectively. It was also determined that AA had little impact on normal cells, as the proliferation rate of the PBMCs was maintained at a steady rate following exposure to various concentrations of AA. Consequently, these results may aid in the development of therapeutic agents for MM. However, the specific mechanism by which AA inhibits cell proliferation remains unknown. Hsu *et al*(12) stated that AA-induced cell growth inhibition in the MCF-7 and MDA-MB-231 cell lines was mediated by the activation of p38 and extracellular signal-regulated kinases 1/2. Additionally, it was demonstrated that a novel mechanism of AA-induced cell death was linked to disruption of the endoplasmic reticulum, with subsequent calcium flux into the cytoplasm (23). AA has been observed to show discrepant anticancer mechanisms in differing cell types, therefore, further scientific experiments and sufficient subsequent proofs are required to resolve these problems.

Tumor cell cycles are closely associated with cell proliferation, which is mainly regulated at two discrete points, including the G_1_/S and G_2_/M phases. In the present study, when the concentration of AA fluctuated in the range of 25–40 μmol/l, the proportion of G_2_/M-phase cells increased from 5.21±2.37 to 54.05±5.66% as the drug dosage increased, indicating that the RPMI 8226 cells were primarily arrested in the G_2_/M phase. Other studies have also reported the AA-induced regulation of tumor cell cycles. For instance, Hsu *et al*(12) stated that AA inhibited cell cycle progression at the S-G_2_/M phase through increasing p21/Cdc2 interaction and decreasing the expression of Cdc2, Cdc25C, cyclin B1 and cyclin A.

To date, the underlying anti-myeloma mechanism of AA remains unclear. FAK is a member of the FAK family of non-receptor protein tyrosine kinases, which resides at sites of integrin clustering and has an important role in cell proliferation, survival and migration (24–28). The increased expression and activity of FAK are frequently correlated with malignant disease and a poor patient prognosis (29–31). Recent studies have indicated that FAK may be a useful therapeutic target for the improved treatment of acute myeloid leukemia cases with poor prognoses (32), and abnormal expression of FAK in patients with MM may be associated with clinical stage and extramedullary infiltration (17). Furthermore, Schmidmaier *et al*(18) concluded that LFA-1/FAK/PI3-K/Akt is a survival pathway in MM and that targeted inhibition may provide new therapeutic options. Notably, the present study also identified that the expression levels of FAK and p-FAK were reduced in AA-treated RPMI 8226 cells. Once again, this study confirmed that AA may serve as a potent anticancer drug and that its mechanism may be associated with the downregulation of FAK expression. Consequently, we speculate that AA may be an adjuvant therapeutic agent for MM and improve the prognosis of high-risk myeloma patients through decreasing the expression of FAK and p-FAK.

In conclusion, AA inhibited cell proliferation by arresting cell cycle progression and downregulating the expression of FAK in the RPMI 8226 cells. These results strongly indicated that AA may be a potential candidate for antitumor therapy, particularly for MM treatment.

## Abbreviations

AA: asiatic acid
MM: multiple myeloma
FAK: focal adhesion kinase
PBMCs: peripheral blood mononuclear cells
